# Asymmetric inheritance of cytoophidia in *Schizosaccharomyces pombe*

**DOI:** 10.1242/bio.20149613

**Published:** 2014-10-31

**Authors:** Jing Zhang, Lydia Hulme, Ji-Long Liu

**Affiliations:** MRC Functional Genomics Unit, Department of Physiology, Anatomy and Genetics, University of Oxford, Oxford OX1 3PT, UK

**Keywords:** Asymmetric inheritance, CTP synthase, *Schizosaccharomyces pombe*, Cytoophidium, Intracellular compartmentation

## Abstract

A general view is that *Schizosaccharomyces pombe* undergoes symmetric cell division with two daughter cells inheriting equal shares of the content from the mother cell. Here we show that CTP synthase, a metabolic enzyme responsible for the *de novo* synthesis of the nucleotide CTP, can form filamentous cytoophidia in the cytoplasm and nucleus of *S. pombe* cells. Surprisingly, we observe that both cytoplasmic and nuclear cytoophidia are asymmetrically inherited during cell division. Our time-lapse studies suggest that cytoophidia are dynamic. Once the mother cell divides, the cytoplasmic and nuclear cytoophidia independently partition into one of the two daughter cells. Although the two daughter cells differ from one another morphologically, they possess similar chances of inheriting the cytoplasmic cytoophidium from the mother cell, suggesting that the partition of cytoophidium is a stochastic process. Our findings on asymmetric inheritance of cytoophidia in *S. pombe* offer an exciting opportunity to study the inheritance of metabolic enzymes in a well-studied model system.

## INTRODUCTION

The budding yeast *Saccharomyces cerevisiae* and the fission yeast *Schizosaccharomyces pombe* are the most studied yeast species, and perhaps the most studied unicellular eukaryotes (Forsburg, 2005). Both yeast species provide excellent models for fundamental biological research. They have been used for the study of various biological processes including cell growth, cell cycle, intracellular trafficking, metabolism, organelles, and cell division (Mitchison, 1957; Mitchison, 1958). While these two species share many features, they have been separated evolutionarily more than 1000 million years ago (Heckman et al., 2001; Hedges, 2002). Perhaps the most notable distinct feature is that *S. cerevisiae* divides, via budding (so it is called budding yeast), while *S. pombe* does fission (so it is called fission yeast). Morphologically budding yeast undergoes asymmetric division. The mother cell keeps most of the cytoplasmic content, while the bud starts with very little cytoplasmic material. The growth of *S. pombe* cells starts with elongating their tips and then divides by binary fission after forming a division septum at the central region. Although it is believe that the two daughter *S. Pombe* cells symmetrically inherited most of their components from the mother cell, there are a few exceptions. For example, the spindle pole bodies appear to be non-equivalent after division and the asymmetry of the spindle pole bodies may be established early during mitosis (Cerutti and Simanis, 1999). *Schizosaccharomyces pombe* are able to switch between two mating types, plus (P) and minus (M). Inheritance of mating type switching is asymmetric due to a strand specific and site-specific imprint. The imprint is a programmed single stranded nick that initiates a recombination event following replication at the *mat1* locus. The break can be repaired using either the transcriptionally silent *mat2P* or *mat3M* as a template for recombination (Klar, 1987).

In 2010, three groups independently discovered that CTP synthase, an essential metabolic enzyme responsible for the *de novo* synthesis of the nucleotide CTP, can form filamentous structures in *Drosophila* (Liu, 2010), bacteria (Ingerson-Mahar et al., 2010) and budding yeast (Noree et al., 2010). This subcellular structure has been termed as the cytoophidium, which means ‘cellular serpent’ in Greek, or CTP synthase filament (Liu, 2010; Liu, 2011). Subsequently, cytoophidia have been found in human cells (Carcamo et al., 2011; Chen et al., 2011). The lineages for bacteria and human have been separated more than 3 billion years ago, yet their CTP synthase molecules can form cytoophidia, suggesting that cytoophidia represent a novel type of organelle which is highly conserved during evolution and natural selection.

Here we report that CTP synthase forms filamentous cytoophidia in the cytoplasm and nucleus of *S. pombe* cells. We show that both cytoplasmic and nuclear cytoophidia are asymmetrically inherited during cell division. In addition, we demonstrate that cytoophidia are highly dynamic. Furthermore, we observe that the cytoplasmic and nuclear cytoophidia independently partition into one of the two daughter cells during cell division. Our quantitative analysis indicates that the partition of cytoophidium is a stochastic process.

## MATERIALS AND METHODS

### *S. pombe* strains

The *cts1^+^* gene was amplified by PCR from genomic DNA with primers containing ApaI and Xhol restriction sites. Following digestion with ApaI and XholI (New England Biolab), the *cts1^+^* fragment was ligated into plasmid pSMUY2+ (supplementary material Fig. S1) using T4 DNA ligase (New England Biolab). The resulting plasmid was termed pSMUY2-cts1 (supplementary material Fig. S2). pSMUY2-cts1 was linearized with SpeI (New England Biolab) and transformed into the wild-type *S. pombe* strain 001 using the lithium acetate method (Paul Nurse Lab Manual) resulting in CTPS-YFP expression from the endogenous promoter at the endogenous locus. The mCherry-Ish1 strain was obtained from Tokuko Haraguchi (Asakawa et al., 2010).

The oligos used for plasmid construction were as follows:

ApaI cts1 fw – tttgggccc CTGTCATGTTGGTCCCGAACAG

XhoI cts1 rv – tttctcgag ACTGATGGTGACGACAGTGGCT

The *S. pombe* strain used for transformation was as follows:

h+; ade6-M216, leu1–32, ura4-D18.

### *S. pombe* cell culture

All the cells were cultured in yeast extract with three supplements adenine, leucine and uracil (YE3S, supplementary material Table S1) at 32°C with a starting OD_600_ value of 0.1. Cell growth was monitored by OD_600_, with a 0.1∼1.0 OD_600_ value indicating exponentially growing cells and over 1.0 OD_600_ value indicating stationary cells.

### Cell fixation and confocal microscopy

For fixed samples, *S. pombe* cells were collected during exponential stage and fixed in 4% paraformaldehyde for 10 min. The fixed cells were washed by PBS and stained with Hoechst 33340. For live imaging, *S. pombe* cells were cultured in glass-bottomed Petri dishes. Imaging of fixed samples or time lapse of live cells were acquired under 63× objective on a laser-scanning confocal microscope (Leica TCS SP5 II confocal microscope).

## RESULTS

### CTP synthase forms cytoplasmic and nuclear cytoophidia in *S. pombe*

**Fig. 1.**
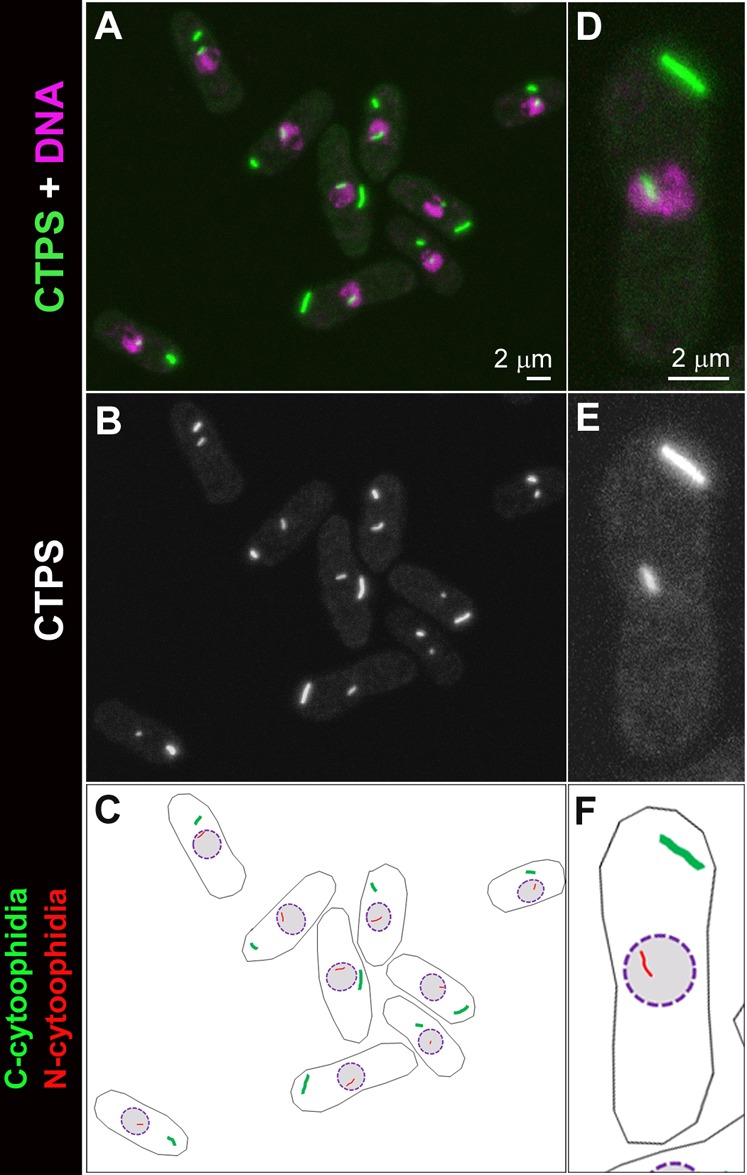
CTP synthase forms cytoophidia in the cytoplasm and nucleus of *S. pombe* cells. (A–C) CTP synthase (CTPS) fused to YFP was expressed under the endogenous promoter. Each of the nine *S. pombe* cells in this field shows a pair of filamentous structures, i.e. cytoophidia. The longer and thicker one localizes in the cytoplasm, while the shorter and thinner one is associated with the nucleus. (D–F) Zoom-in image of one *S. pombe* cell, which contains one C-cytoophidium and one N-cytoophidium. Scale bars: 2 µm.

To study cytoophidia systematically, we decided to investigate CTP synthase in a well-studied single-celled organism, *S. pombe*. In *Drosophila*, we have found that YFP fused at the C-terminus of CTP synthase mimics the distribution pattern of endogenous CTP synthase (Azzam and Liu, 2013). To control the expression of CTP synthase-YFP, we used endogenous promoter to express CTP synthase-YFP at the endogenous locus in *S. pombe*. As expected, we observed that CTP synthase-YFP could form filamentous structures in *S pombe*. Closely looking at the fission yeast cells, we found that CTP synthase-YFP could form cytoophidia both in the cytoplasm and in the nucleus (Fig. 1). Since the strain that we constructed with CTP synthase-YFP grew normally, we assume that the CTP synthase-YFP protein is functional as it is the only copy of CTP synthase, an essential gene in *S. pombe*.

**Fig. 2.**
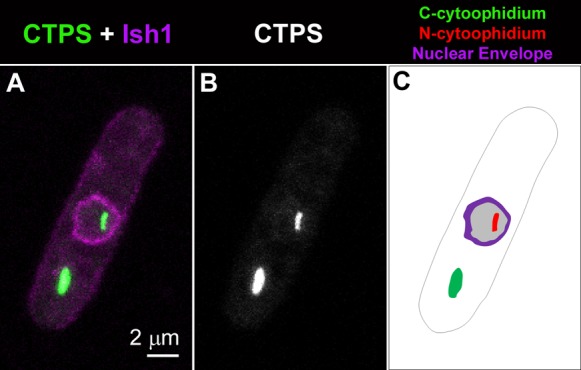
N- and C-cytoophidia reside at opposite sides of the nuclear envelope. CTP synthase (CTPS) fused to YFP was expressed under the endogenous promoter. Ish1, a marker for the nuclear envelope, was fused to mCherry. Scale bar: 2 µm.

To better detect the relative distribution of cytoophidia, we generated a strain expressing CTP synthase-YFP and mCherry-Ish1, a marker for the nuclear envelope. We observed that N-cytoophidia resided at the inner side of the nuclear envelope, while C-cytoophidia resided at the outer side of the nuclear envelope (Fig. 2). These results confirm that the CTP synthase molecules are compartmentalised into at least two distinct pools and that N-cytoophidia and C-cytoophidia are separated by the nuclear envelope in *S. pombe*.

### Cytoophidia are inherited asymmetrically during cell division

**Fig. 3.**
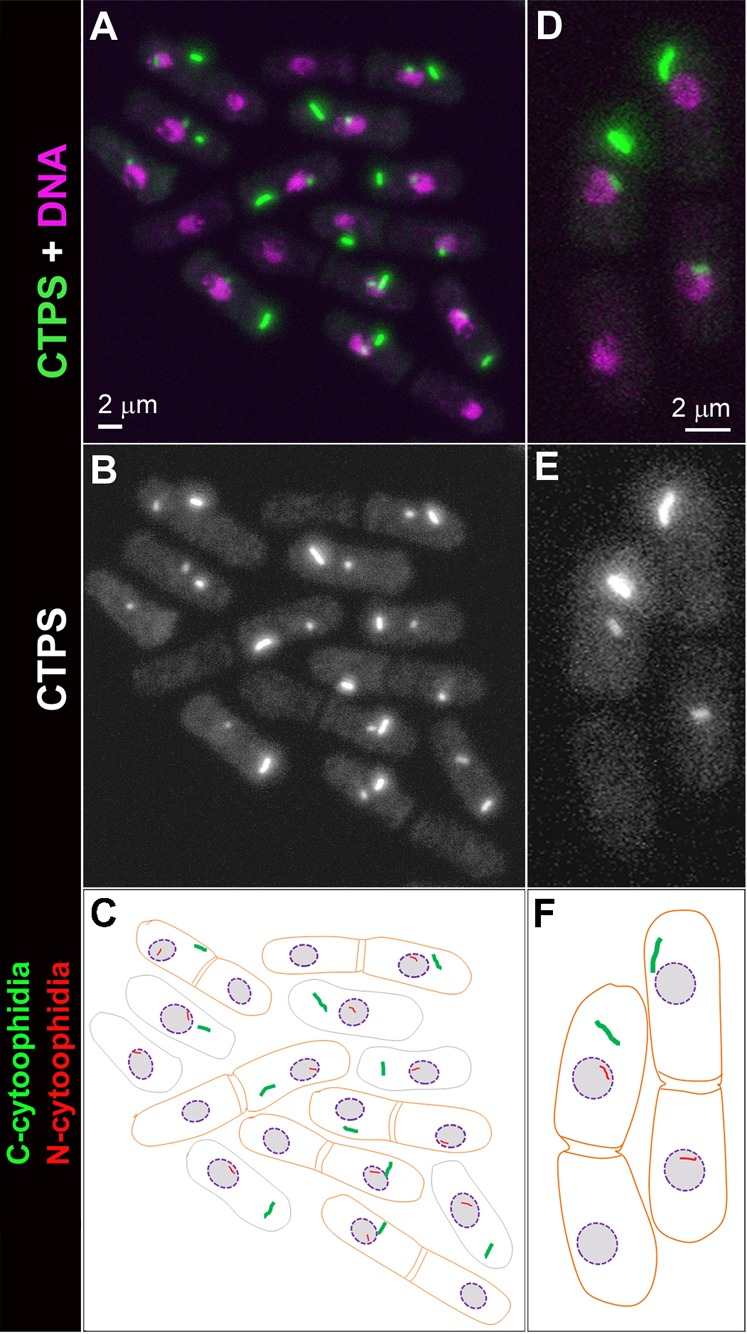
Differential inheritance of cytoophidia in *S. pombe*. CTP synthase (CTPS) fused to YFP was expressed under the endogenous promoter. (A–C) Among the six pairs of cells (outline in orange in panel C), only one cell in each pair contains a C-cytoophidium, and only one cells in each pair contains a N-cytoophidium. In many cases, the cell with a C-cytoophidium also contains an N-cytoophidium. In a pair of cells (labeled with a star) that one cell contains a C-cytoophidium, while the other cell possesses an N-cytoophidium. (D–F) Zoom-in image of two pairs of *S. pombe* cells showing that both C- and N-cytoophidium are differentially inherited. Scale bars: 2 µm.

**Fig. 4.**
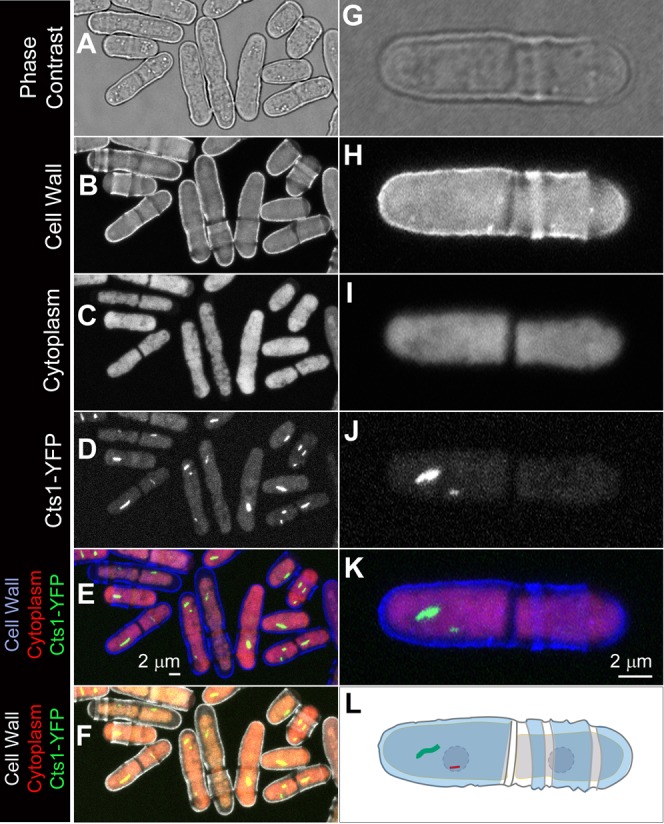
*S. pombe* cells are morphologically asymmetric and polarized. (A–F) A group of *S. pombe* cells at different phases of cell cycle. The difference between the two poles can be appreciated when the cell wall (B) and the cytoplasm (C) are outlined by Calcofluor white staining. (G–L) A pair of *S. pombe* cells. These two cells are morphologically distinct from one another. In these panels, the cell on the left inheriting the old end (‘Smooth cell’) also inherits the C- and the N-cytoophidium, while the other cell (‘Isis Rough cell’) inherits neither cytoophidia. Scale bars: 2 µm.

Next we analysed in the frequencies and numbers of C- and N-cytoophidia in *S. pombe* cells. We found that the most majority of *S. pombe* cells have one C-cytoophidium and one N-cytoophidium. This raises a fundamental issue: how C- and N-cytoophidia are passed into the next generation after cell division. *S. pombe* has been a popular system for studies of cell growth and division due to its regular shape and size. As in most systems, the central events of *S. pombe* cell reproduction are chromosome replication in S phase, followed by chromosome segregation and nuclear division (mitosis) and cell division (cytokinesis) in M phase. While *S. cerevisiae* has an extended period at the gap between M and S phases (G1), *S. pombe* remains in G2 phase of the cell cycle for an extended period (Hartwell et al., 1974; Mitchison, 1972; Nurse et al., 1976). In *S. pombe*, two daughter cells remain associated with each other physically after mitosis and cytokinesis at G1/S phases. The abscission of the paired cells occurs afterwards. We analyzed cells at G1/S phase when two daughter cells are still clearly associating with each other (after cytokinesis but before scission) and observed that the distributions of C- and N-cytoophidia in one cell differ from those in another cell. After *S. pombe* cells undergo cell division, we can see only one out of the two daughter cells has C-cytoophidia (Fig. 4). In 99.5% cells (n = 358), we observed that C-cytoophidia can be detected in only one of the two daughter cells or the C-cytoophidia in one daughter cell are dramatically larger than those in the other cell (supplementary material Fig. S3). Similarly, N-cytoophidia are only detectable in one of these two daughter cells. These data suggest that both C- and N-cytoophidia are differentially inherited during *S. pombe* cell division. Interestingly, N-cytoophidium is not always inherited by the same cell that inherits the C-cytoophidium (Fig. 3).

### Cytoophidium inheritance is a stochastic process

Calcofluor white staining has been widely used for labeling the cell wall of *S. pombe* cells. Using 405 nm laser as the excitation beam, we could detect the cell wall when collecting the emission signal at 420–470 nm (Fig. 4A,B) and detect the cytoplasm when collecting the emission signal at 620–670 nm (Fig. 4C). The birth scars are seen as dark rings crossing the *S. pombe* cell wall (Fig. 4B). Since the old end has much higher growth rate than the new end, the distance between the old end and the birth scar arising from the last cell division will be much larger than that between the new end and the birth scar.

When we looked at cytoophidia along with other markers for the cell wall and cytoplasm, we observed that C-cytoophidia have the preference to be at the periphery the *S. pombe* cells, making the cytoophidium localises closer to one end than another (Fig. 4D–F). In order to better understand cytoophidium inheritance in *S. pombe*, we focused on cells in pairs for further analysis. After mitosis and cytokinesis, the paired cells are still physically associated for a certain period before the abscission occurs. Each of the two daughter cells inherits one end of the mother cells. To distinguish these two daughter cells, we refer them as the ‘Smooth cell’ for the cell inheriting the old end of the mother and the ‘Rough cell’ for the one inheriting the new end of the mother cell (Fig. 4G–L). Since the old end of the mother cell grows extensively before the cell division, the Smooth cell would be mostly free from birth scars (Fig. 4H). In contrast, the Rough cell contains one or a few birth scars from previous generations.

Given the morphological differences between the two daughter cells, we next asked whether the cytoophidium is differentially inherited by one of the two daughter cells. We focused on C-cytoophidia because of their large sizes. Our quantificational analysis showed that the chances of inheriting the C-cytoophidia is similar between the Smooth cell and the Rough cell (49.4% vs. 50.6%, n = 178). These data suggest that the inheritance of C-cytoophidia is stochastically passed into one of the two daughter cells.

### Both cytoplasmic and nuclear cytoophidia are highly dynamic

**Fig. 5.**
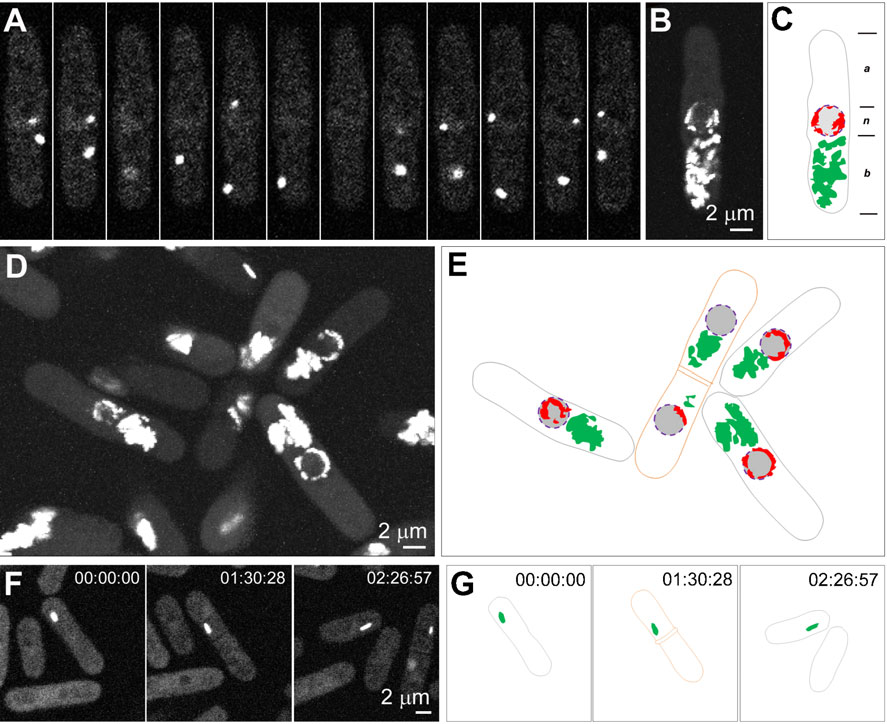
Cytoophidia are dynamic and constrained in *S. pombe*. (A–E) Live *S. pombe cells* are recorded every 5 sec for 10 min. CTP synthase (CTPS) fused to YFP was expressed under the endogenous promoter. (A) 12 snapshots of a cell. Maximum projections of 118 frames recorded in 10 min for a cell (B) or a group of cells (D). The movement of the C-cytoophidium, traced in green (C,E), appears to be constrained in certain area in the cytoplasm, while the N-cytoophidium, traced in red (C,E), appears to be constrained at the periphery of the nucleus. (F,G) Snapshots of live imaging of cells undergoing division. Scale bars: 2 µm.

**Fig. 6.**
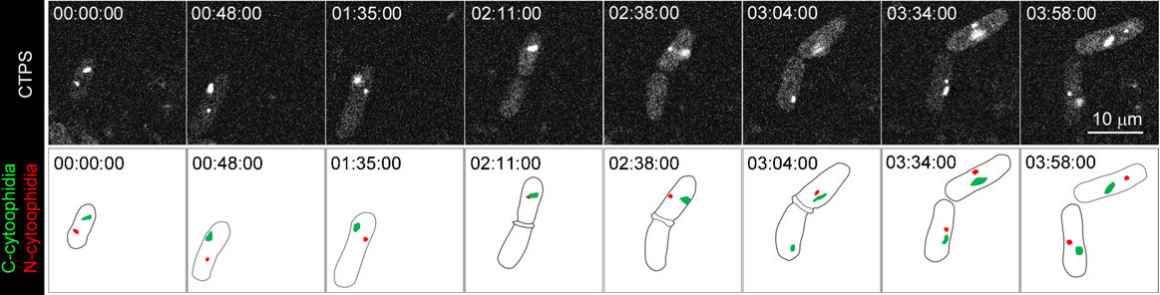
*De novo* formation of cytoophidia in *S. pombe*. Snapshots of a 4-hour live imaging recording a cell undergoing cell division. CTP synthase (CTPS) fused to YFP was expressed under the endogenous promoter. At the start timepoint (00:00:00), the mother cell clearly shows one cytoplasmic cytoophidium (C-cytoophidium, red in the cartoons) and one nuclear cytoophidium (N-cytoophidium, green in the cartoons). During cell division (02:11:00), the daughter cell at the top inherits the cytoophidia. The cell at the bottom does not have cytoophidia for about 50 min until a cytoophidium forms *de novo* (03:04:00). At the timepoint 03:34:00, both C- and N-cytoophidia inside the cell at the bottom grow, similar to the cytoophidia inside the cell at the top. Scale bar: 10 µm.

To better understand the behavior of cytoophidia, we performed live imaging with *S. pombe* cells expressing Cts1-YFP. When we recorded live cells every 5 sec, we found that N-cytoophidia appeared highly dynamic. The traces of N-cytoophidia indicate that they mostly stay at the periphery of the nucleus, raising the possibility that N-cytoophidia tether on the nuclear envelope. Traces of C-cytoophidia showed that they moved dynamically in the cytoplasm between one end and the central region when the nucleus resides. The nucleus appears to serve as a barrier which, in most cases, prevents the C-cytoophidium moving from one end towards another end of the cell (Fig. 5A–E; supplementary material Movies 1 and 2). To see if the confinement of C-cytoophidia at G2 phase affects cytoophidium during cell division, we performed live imaging of *S. pombe* cells processing from G2 to mitosis, cytokinesis and abscission. Indeed, we found that the location of cytoophidia at G2 phase correlated with the differential inheritance of cytoophidia (Fig. 5F,G; supplementary material Movie 3). Since we found that more than 90% *S. pombe* cells contain C-cytoophidia, we predicted that cytoophidia will form *de novo* in the daughter cell that does not inherit the C-cytoophidium from the mother cell. Our prediction was confirmed by long-period time-lapse studies (Fig. 6; supplementary material Movie 4).

## DISCUSSION

### Functions of cytoophidia

Recently, many metabolic enzymes have been found to form cytoophidium-like filaments, suggesting intracellular compartmentation of metabolic pathways is more general than previously thought. A large-scale screen of yeast GFP library identified that over 20% of strains examined form distinct intracellular structures (Narayanaswamy et al., 2009). A second screen of 40% GFP collection in budding yeast showed 9 proteins form 4 types of filamentous structures (Noree et al., 2010). More recently, several studies demonstrated that polymerization of CTP synthase into cytoophidia downregulates enzymatic activity in bacteria (Barry et al., 2014), budding yeast (Noree et al., 2014), fruit flies and human cell lines (Aughey et al., 2014). Thus cytoophidium formation seems to be a general strategy to regulate enzymatic activity. The assembly and disassembly of the cytoophidium and its kinds provide a swift and robust buffer mechanism to maintain cellular homeostasis during development and in mediating adaptive metabolic responses (Aughey et al., 2014; Barry et al., 2014; Noree et al., 2014; Petrovska et al., 2014).

### Nuclear cytoophidia

Recently we have reported that CTP synthase can form both cytoplasmic cytoophidia (C-cytoophidia) and nuclear cytoophidia (N-cytoophidia) in mammalian cells (Gou et al., 2014). Our results in *S. pombe* and mammalian cells suggest that both C- and N-cytoophidia are evolutionarily conserved.

Our live imaging study suggests that N-cytoophidia are mostly localise at the periphery of nuclei in *S. pombe*. Would it be possible that N-cytoophidia are tethered at the inner side of the nuclear envelope? Gitai and colleagues have found that CTP synthase functionally interacts with the intermediate filament crescentin in curved bacteria *Caulobacter crescentus* (Ingerson-Mahar et al., 2010). It is of interest to see if N-cytoophidia are functionally linked to the nuclear lamina, which is composed of intermediate filaments and membrane associated proteins.

### Dynamics of cytoophidia

Cytoophidia are highly dynamic by several criteria. Here we show that both C-cytoophidia and N-cytoophidia undergo constrained movement in *S. pombe* cells. In addition, we demonstrate that cytoophidia assemble *de novo* in one daughter cell after mitosis. In *C. crescentus*, CTP synthase molecules form from very small foci into 500-nm long filaments in stalked cells (Ingerson-Mahar et al., 2010). Finally, our previous study in mammalian cells indicates that smaller micro-cytoophidia can undergo multiple rounds of fusion to form larger macro-cytoophidia (Gou et al., 2014).

Multifaceted dynamics of cytoophidia reflect the complexity of the biogenesis of these structures. It remains to be determined whether cytoophidium movement in the cytoplasm is based on microtubules and/or actin filaments. Cytoophidia may be moving around together with other organelles. It is not inconceivable that movement of cytoophidia serves as an efficient way to transport concentrated enzymes to certain areas. Formation and growth of cytoophidia provide a quick response to environmental stress. Equilibrium between micro- and macro-cytoophidia through fusion-fission process might add another layer of regulation for metabolic pathways.

### Asymmetries of *S. pombe* cells

*S. pombe* cells have been considered being asymmetric but unpolarised (Macara and Mili, 2008). Under phase contrast, *S. pombe* cells can be seen as rod-shaped and typically measure 3–4 µm in diameter and 7–14 µm in length (Fig. 5). *S. pombe* is asymmetric in the sense that its cell shape is cylindrical with a long axis and a short axis. Although it was assumed that the two ends of the cylinder are identical, multiple lines of evidence show that the two ends of *S. pombe* are different from one another. First, each *S. pombe* cell has one old end, which was inherited from the dividing mother cell, and one new end arising from cell division. Second, the growth timepoint and growth rate are different for the two ends. Wild-type *S. pombe* cells extend exclusively at their two ends, increasing the length of their rod-shaped bodies while keeping similar widths. After cell division, *S. pombe* starts to grow only at old ends for a while before the new ends initiate growth at a point in G2 known as new end take off (NETO) (May and Mitchison, 1995; Mitchison and Nurse, 1985). Third, the cell wall associated with the two ends has different patterns, especially with birth or division scars, which mark the position of a previous septum site (Mitchison and Nurse, 1985). Birth scars are not only obvious under scanning electron microscopy and transmission electron microscopy, they are easily detected when the *S. pombe* cells were stained by a fluorescence dye Calcofluor white. In addition, spindle pole bodies have been found to be non-equivalent during mitosis and remain so throughout interphase (Cerutti and Simanis, 1999).

In this study we show that cytoophidia are also inherited asymmetrically during cell division. We also find that the inheritance of cytoophidia does not seem to have preference to the ends of the mother cell. It would be interesting to see whether additional cellular components and fate determinants are asymmetrically inherited in *S. pombe* and if yes, how their differential inheritance is determined. Further studies are required to investigate whether the asymmetric inheritance is linked with the asymmetry of the spindle pole bodies. However, it remains to be determined whether there is a mechanism that causes asymmetric segregation of cytoophidia, since C-cytoophidium and N-cytoophidium only have one each in a mother cell. It is possible that the septum itself by default will segregate them asymmetrically without any additional mechanism.

Asymmetric cell division has been suggested to be a widespread strategy for cellular organisms to restrict senescence to one daughter (Macara and Mili, 2008). However, it remains a question whether fission yeast cells undergo asymmetric cell division. Asymmetric segregation of large aggregates induced by stress seems to contribute to aging in *S. pombe* (Coelho et al., 2013). Whether and how the differential inheritance of cytoophidia and other cellular components contribute to fitness and aging are left for future studies.

## Supplementary Material

Supplementary Material
