# Supplementary material for: Asymmetric inheritance of cytoophidia in Schizosaccharomyces pombe
Source: Biol Open. 2014 Oct 31;3(11):1092–7. doi: 10.1242/bio.20149613 (PMC4232767; doi:10.1242/bio.20149613)
Supplement: Supplementary Material [file supp_3_11_1092__index.html]

Asymmetric inheritance of cytoophidia in Schizosaccharomyces pombe — Asymmetric inheritance of cytoophidia in Schizosaccharomyces pombe — Supplementary Material 

# Asymmetric inheritance of cytoophidia in *Schizosaccharomyces pombe*

## bio.20149613 Supplementary Material

**Files in this Data Supplement:**

- Supplementary Material - Jing Zhang et al. doi: 10.1242/bio.20149613
- Movie 1 - **Movie 1. Time lapse of a *S. pombe* cell.** Duration: 10 minute; recorded approximately every 5 seconds. Related to Fig. 3A–C.
- Movie 2 - **Movie 2. Time lapse of a group of *S. pombe* cells.** Duration: 10 minutes duration; recorded approximately every 5 seconds. Related to Fig. 3D,E.
- Movie 3 - **Movie 3. Time lapse of a *S. pombe* cell undergoing cell division.** Duration: 2 hours 33 minutes; recorded approximately every 2 minutes. Related to Fig. 3F,G.
- Movie 4 - **Movie 4. Time lapse of a *S. pombe* cell undergoing cell division.** Duration: 4 hours; recorded approximately every minute. Related to Fig. 4.
