# Supplementary material for: Asymmetric inheritance of cytoophidia in Schizosaccharomyces pombe
Source: Biol Open. 2014 Oct 31;3(11):1092–7. doi: 10.1242/bio.20149613 (PMC4232767; doi:10.1242/bio.20149613)
Supplement: Supplementary Material [file supp_bio.20149613_bio.20149613-s1.pdf]

Biology Open (2014) 000, 1–6 doi:10.1242/bio.20149613

Supplementary Material

Jing Zhang et al. doi: 10.1242/bio.20149613

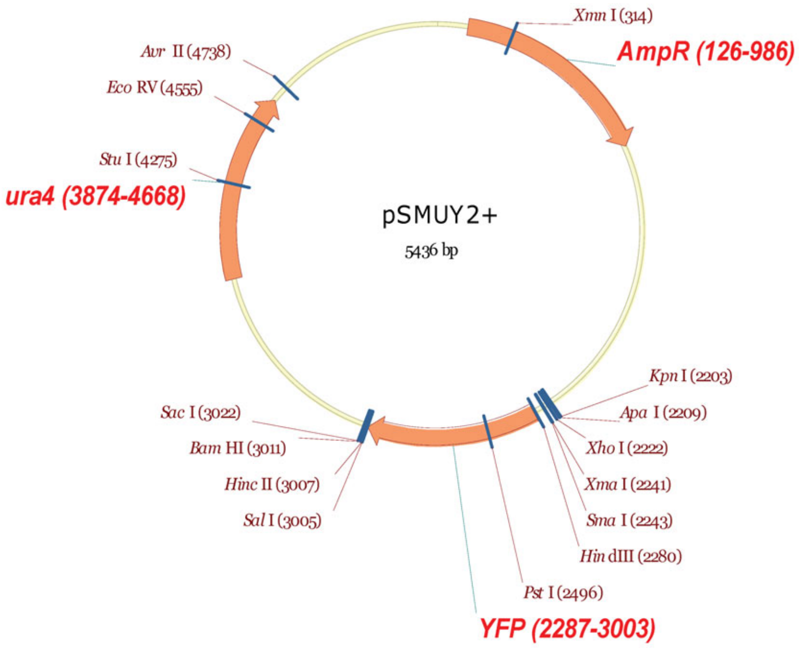

Fig. S1. Backbone plasmid pSMUY2+.

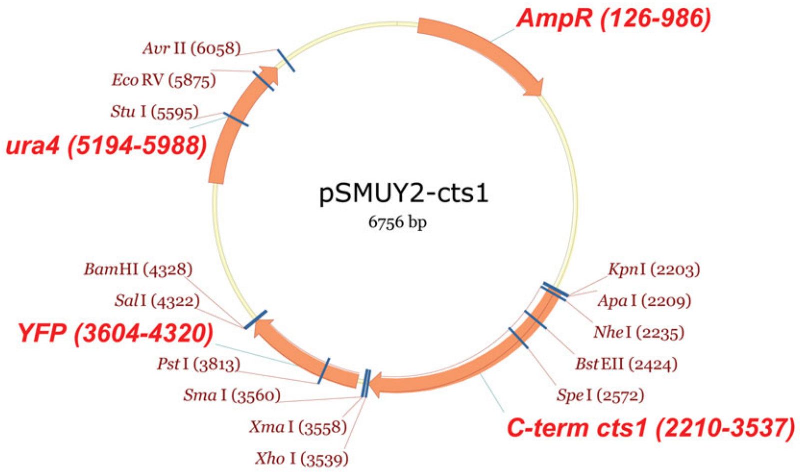

Fig. S2. Plasmid pSMUY2-cts1.

Biology Open

Biology Open (2014) 000, 1–6 doi:10.1242/bio.20149613

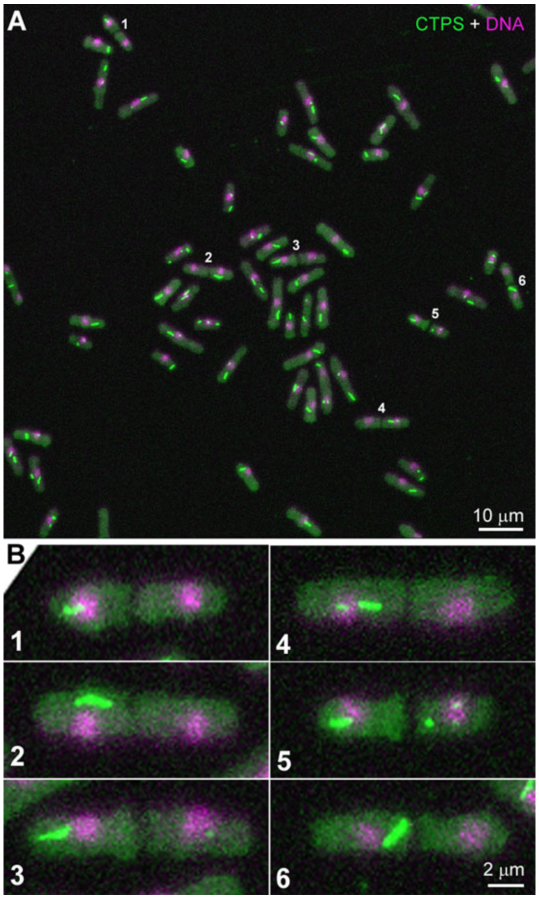

Fig. S3. CTP synthase forms cytoophidia in *S. pombe*. (A) Cytoophidia are labeled by CTP synthase-YFP (CTPS, green). Most cells in this field contain both cytoplasmic and nuclear cytoophidia. Six pairs of cells are labeled by numbers. (B) Zoom-in images of the six paired cells in panel A. Note the different pattern of cytoophidia between the two cells in each pair. The zoom-in images have been rotated so the cells on the left hand side contain large cytoophidia in the cytoplasm, while the cells on the right hand side are either lack of cytoplasmic cytoophidia or only contain small-sized cytoophidia. Scale bars: 10 μm (A), 2 μm (B).

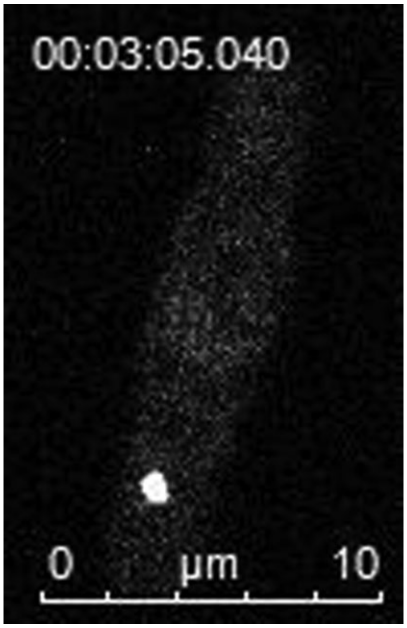

Movie 1. Time lapse of a *S. pombe* cell. Duration: 10 minute; recorded approximately every 5 seconds. Related to Fig. 3A–C.

Table S1. The recipe for YE3S*, a standard rich medium we use for growing *S. pombe*

Yeast extract	5 g/L
Glucose	30 g/L
Adenine	225 mg/L
Leucine	225 mg/L
Uracil	225 mg/L

*3 stands for the three supplements including adenine, leucine and uracil.

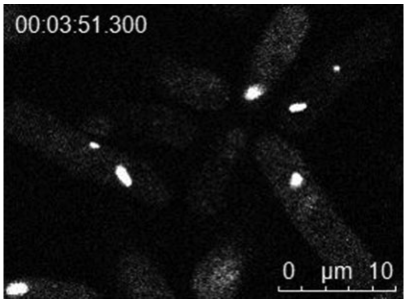

Movie 2. Time lapse of a group of *S. pombe* cells. Duration: 10 minutes duration; recorded approximately every 5 seconds. Related to Fig. 3D,E.

Biology Open (2014) 000, 1–6 doi:10.1242/bio.20149613

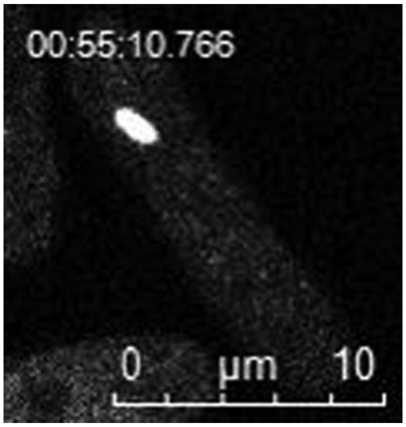

Movie 3. Time lapse of a *S. pombe* cell undergoing cell division. Duration: 2 hours 33 minutes; recorded approximately every 2 minutes. Related to Fig. 3F,G.

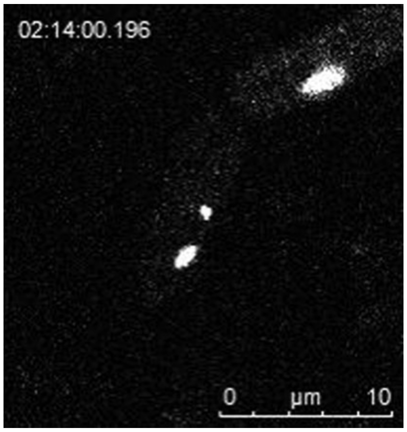

Movie 4. Time lapse of a *S. pombe* cell undergoing cell division. Duration: 4 hours; recorded approximately every minute. Related to Fig. 4.
